# Multi-Component Exercise with High-Intensity, Free-Weight, Functional Resistance Training in Pre-Frail Females: A Quasi-Experimental, Pilot Study

**DOI:** 10.14283/jfa.2020.13

**Published:** 2020-03-16

**Authors:** N.W. Bray, G.J. Jones, K.L. Rush, C.A. Jones, Jennifer M. Jakobi

**Affiliations:** 1 School of Health and Exercise Sciences, Faculty of Health and Social Development, University of British Columbia Okanagan, V1V 1V7, Kelowna, British Columbia, Canada; 2 School of Nursing, Faculty of Health and Social Development, University of British Columbia Okanagan, Kelowna, British Columbia, Canada; 3 Southern Medical Program, Faculty of Medicine, University of British Columbia Okanagan, Kelowna, British Columbia, Canada

**Keywords:** Older age, resistance training, muscle strength, quality of life, females

## Abstract

**Background:**

No study has performed an exercise intervention that included high-intensity, free-weight, functional resistance training, and assessed frailty status as an inclusion criteria and outcome measure via original, standardized tools, in pre-frail females.

**Objectives:**

Determine if the intervention strategy is not only feasible and safe, but can also improve frailty status, functional task performance, and muscle strength.

**Design:**

Pilot, quasi-experimental.

**Setting:**

Community.

**Participants:**

20 older-adults with pre-frailty characteristics

**Intervention:**

12-weeks (3 days/week, 45–60 minutes/session) of multi-component exercise, inclusive of aerobic, resistance, balance and flexibility exercises. The crux of the program was balance and resistance exercises, the latter utilized high-intensity, free-weight, functional resistance training. The control group maintained their usual care.

**Measurements:**

1) Feasibility and safety (dropout, adherence, and adverse event)
2) Frailty (Frailty Phenotype, Clinical Frailty Scale, and gait speed)
3) Functional task performance (grip strength and sit-to-stand time); and
4) Isometric and isotonic strength of the knee extensors and elbow flexors.

**Results:**

No participants dropped out of the intervention or experienced an adverse event, and adherence averaged 88.3%. The exercise group became less frail, whereas the control group became more frail. There was a significant within-group improvement in exercise participants gait speed (p ≤ 0.01, +0.24 m/sec), grip strength (p ≤ 0.01, +3.9 kg), and sit-to-stand time (p ≤ 0.01, -5.0 sec). There was a significant within-group improvement in exercise participants knee extension isometric torque (p ≤ 0.05, +7.4 Nm) and isotonic velocity (p = ≤ 0.01, +37.5 °/sec). Elbow flexion isotonic velocity significantly declined within the control group (p ≤ 0.01, -20.2 °/sec) and demonstrated a significant between-group difference (p ≤ 0.05, 40.73 °/sec) post-intervention.

**Conclusions:**

The intervention strategy appears to be feasible and safe, and may also improve frailty status, functional task performance, and muscle strength. These results help calculate effect size for a future randomized controlled trial.

## Introduction

Exercise is considered a suitable therapy to reverse frailty (1, 2, 3) but it is difficult to determine which characteristics of an exercise program are most effective (4, 5). Most exercise intervention studies do not adequately assess the level of frailty (4), convoluting the understanding of positive adaptation and limiting the potential to apply spectrum specific exercise recommendations suggested in a recent literature review (1).

Frailty is also a dynamic process where transitions are subtle (6, 7), bidirectional (8), and sex-dependent; females experience frailty differently than males, succumbing to the syndrome earlier yet living longer (9, 10). Older females also represent a larger percentage of the aging demographic (11), and pre-frail females outnumber those that are frail (12, 13). Thus, there is considerable need to identify exercise interventions that would improve frailty status in this large and growing cohort;

A scoping review identified only 15 exercise studies that used a frailty identification tool to classify participants' frailty status at the onset and conclusion of an intervention (14). However, only five studies (15, 16, 17, 18, 19) included a clearly defined pre-frail sample population and only one focused exclusively on females, which were identified via a modified frailty assessment tool (17). These studies generally utilized higher-repetition, low-intensity, single-joint resistance training exercises. To-date, no study has utilized validated frailty identification tools as both an inclusion criteria and outcome measure to assess an exercise intervention that incorporates high-intensity, free-weight, functional resistance training, exclusively in pre-frail females. Despite a lack of evidence supporting such claim, this may be due to the commonly held belief that such resistance training exercises are unsafe (20).

We performed a pilot study to determine if a multi-component exercise (MCE) program, inclusive of high intensity, free-weight, functional resistance training, is not only feasible and safe, but can improve frailty status, functional task performance, and measures of strength in pre-frail females. It was hypothesized that the exercise group (EX) would improve frailty status, functional task performance, and muscle strength, in comparison to a control group (CON).

## Methods

### Experimental Overview

**Figure 1:**
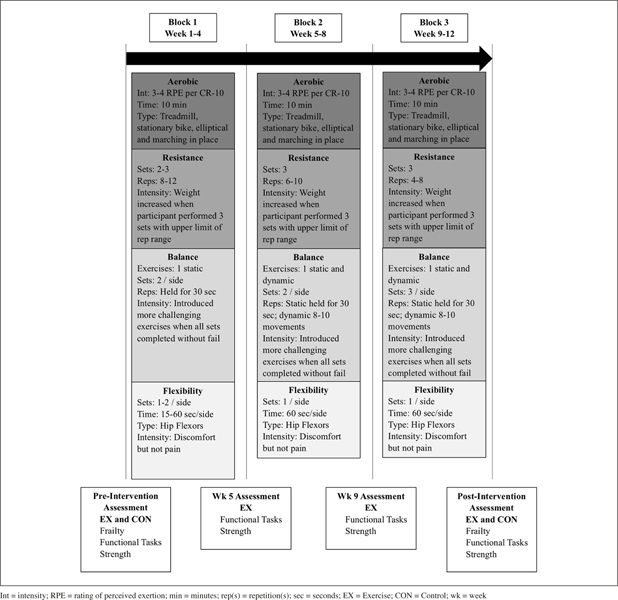
Overview of the multi-component exercise (MCE) intervention and assessments. Similar to resistance training, participants began balance training at different levels and progressed based upon individual ability. The balance training was progressed when the upper limit for time or repetition range was achieved without loss of balance. Training sessions concluded with a flexibility training, utilizing just a kneeling hip flexor flexibility exercise (30). Participants started by stretching for 15 seconds (sec) per leg, and added 15 sec each week, up to a maximum of 60 sec, for a total time of 2 min

EX participants completed a 12-week exercise intervention (3-days/week, 45–60 min/session). The CON maintained their normal routine for the same duration. Participants of both groups were assessed for frailty status, functional task performance, and muscle strength at week 0 (pre-intervention) and 13 (post-intervention), and the latter two measures were repeated at weeks 5 and 9 for the EX (Figure 1).

### Subjects

Participants were recruited from the community. Inclusion criteria consisted of: 1) Females ≥ 65 years of age; 2) A Montreal Cognitive Assessment (21) score ≥ adjusted normative values (22); 3) No contraindications to exercise, as determined by the Physical Activity Readiness–Questionnaire Plus; 4) No major injuries/surgeries to the dominant arm or leg in the last six months; 5) Fluent in English; and 6) A pre-frail frailty status.

Initial recruitment found 53 older adults interested but only 21 agreed to complete the pre-intervention assessment, with one being excluded. As a quasi-experimental study, participants were randomized to the EX and CON based upon their perceived availability to participate in exercise. Current interventions utilizing exercise require 80% adherence (23). As a result, 9/20 participants were placed in the EX.

All participants read and signed a letter of informed consent. Ethical approval was granted by the institutional Research Ethics Board (H16-00712). All experimental procedures involved in the study conformed to the declaration of Helsinki.

### Multi-Component Exercise (MCE)

Kinesiology students led each session. Details of the aerobic warm-up, as well as the balance exercises and flexibility cool-down are within Figure 1. Resistance training was divided into three blocks of four weeks; each new block decreased the number of repetitions to be completed and subsequently increased the resistance, thus, transitioning from training muscular endurance to strength. Within each block, the resistance was also increased when participants reached the upper limit of the repetition range for all sets. Rest periods between sets ranged from 1-3 minutes. To aid progressive overload, participants disclosed their rating of perceived exertion after the last set of every exercise. Details about the training principles can be found in our companion article, “Practical Implications for Strength and Conditioning of Older Pre-Frail Females” (24).

Four free-weight resistance exercises were selected: 1) Squat; 2) Deadlift; 3) Bench Press; and 4) Leg Press. Exercises 1-3 replicate functional movements, such as standing from a toilet, opening a door, and picking up groceries from the ground; these exercises utilized dumbbells (Hex Dumbbell, Northern Lights Inc, Cornwall, ON) and/or barbells (The Bella 2.0 — Females's Bar, Rogue Fitness; Columbus, OH) with weighted plates (Virgin Rubber Grip Olympic Plates, Element Fitness; Latvia). The fourth exercise supplemented the Squat exercise but was less technically demanding, and utilized an incline leg press machine (TuffStuff PPL-960 45°Leg Press, TuffStuff Fitness Equipment, Chino, CA). Each session started with the squat or bench press and finished with the deadlift or inclined leg press.

### Experimental Assessments

Feasibility and Safety: Participant dropout and adverse events were recorded in an ongoing electrical document log. Adherence rates were maintained in an ongoing paper log that participants were required to sign upon arrival to each intervention session.

Frailty Status: Participants were included in this study if they were classified as pre-frail according to guidelines in at least one of the following three tools:

1. 1-2 on the frailty phenotype (FP) (25)
2. 4-6 on the clinical frailty scale (CFS) (26)
3. Gait speed (GS) of ≥ 1.0 – < 1.5 m/sec indicated pre-frailty (27)

Previous research suggests that several frailty tools may provide a more reliable measure of frailty status, and that the FP and CFS do not always provide the same classification (9, 28). Therefore, GS was used as a third frailty criterion.

Functional Task Performance: GS was assessed across 8 meters (m), excluding acceleration (2m) and deceleration (2m) zones, at a self-selected walking speed that was considered normal. The GS test was completed twice and the fastest trial was used for data analysis. Handgrip strength was measured while standing with a dynamometer (Baseline Smedley, Fabrication Enterprises Incorporated, White Plains, NY) held at arm's length and slightly abducted. The dynamometer was squeezed as hard as possible for three seconds, and completed twice for each hand. The order of grip testing was randomized and trials occurred between gait tests. The highest score was used for both functional task performance and FP. The sitto-stand (STS) task was adopted from the short physical performance battery (29).

Muscle Strength Performance: The Biodex Dynamometer System 4 Pro (Biodex Medical Systems Incorporated, Shirley, NY) was used to assess peak torque/velocity of isometric/isotonic knee extension (KE) and elbow flexion (EF). For KE and EF, participants were seated with their hips flexed to ~100°. Restraining straps crossed the chest and opposite hip, and for KE, a third was secured across the thigh of the tested leg. For KE, the lateral femoral condyle of the dominant leg was aligned with the dynamometer center of rotation. To account for the effect of gravity, the weight of the limb was calculated by the Biodex with the knee extended to 160°. For EF, the medial epicondyle of the elbow was aligned with the dynamometer center of rotation. The shoulder was slightly forward flexed (10-15°) and abducted. Positioning was measured, recorded, and replicated for all sessions.

Isometric KE and EF contractions were executed at a joint angle of 90°. Isotonic KE was also initiated from 90° but EF isotonic contractions were initiated from 160°. Range of motion of isotonic contractions was 90-160° for KE and the inverse for EF. Isotonic resistance was 20% of the peak torque from the strongest isometric contraction of the pre-intervention assessment. Participants completed five isometric and isotonic contractions for both KE and EF, with two minutes of rest between contractions. All contractions were recorded and the highest value was used for data analysis. The analogue signal was sampled at 2,000 Hz and stored for offline analysis using the Biodex software.

### Statistical Analysis

Pre-intervention characteristics were compared using an independent sample t-test. A sample size calculation for a 2-tailed study design advised a minimum sample size of six subjects per group to attain a statistical power of 0.80 for the KE strength variable. A two-way mixed ANOVA of time by group assessed all outcomes. A one-way ANOVA was used to evaluate any significant interactions and main effects. The EX was also examined over time using a one-way repeated measures ANOVA, with a Bonferroni post-hoc test. Sphericity was assessed (Mauchly's) and a Greenhouse-Geisser correction applied if violated (p < 0.05). Data that violated normality and homogeneity of variances was transformed using the function Log10. Statistical significance was p ≤ 0.05. All values are reported as mean ± standard deviation (SPSS Statistics V.24, IBM Canada Ltd. Markham, Ontario).

## Results

Characteristics: Participant characteristics were similar between groups (Table 1). Three CON participants were removed from the analysis because they were unable to complete the post-intervention assessment at the required date due to a fall-related injury unrelated to the study (n = 2) or illness (n = 1). No EX participants dropped out or experienced an adverse event during the intervention, and intervention adherence averaged 88.3%. One EX participant completed <65% of exercise sessions (seasonal flu) and was subsequently removed from the final analysis. A shoulder injury, unrelated to the exercise program, precluded one EX participant from completing the grip strength, STS, and EF measures at week 13.

**Table 1:** Participant characteristics (mean ± SD)

Characteristics	CON	EX
	n = 8	n = 8
Age (years)	72.4 ± 5.4	72.9 ± 4.8
Height (cm)	156.4 ± 7.7	162.5 ± 4.0
Weight (kg)	67.1 ± 11.0	75.8 ± 23.3
BMI (kg/m^2^)	27.4 ± 4.0	28.6 ± 8.5
MoCA	25.6 ± 2.6	25.0 ± 3.0
Comorbidities per Participant	1.9 ± 1.5	2.1 ± 1.2


SD = standard deviation; CON = control; EX = exercise; BMI = body mass index; MoCA = Montreal Cognitive Assessment; cm = centimeters; kg = kilograms; kg/m^2^ = kilograms per meter squared.

**Figure 2:**
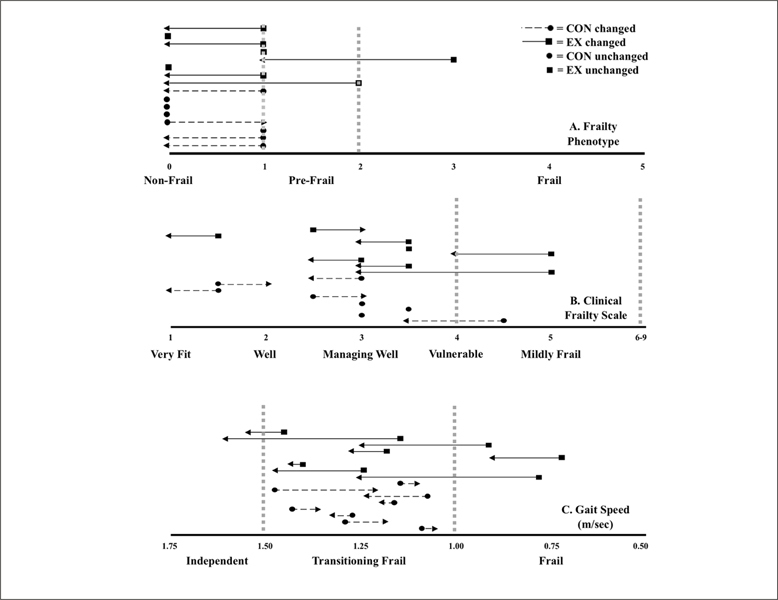
Changes in frailty status across all three frailty identification tools: A) Frailty Phenotype (FP), n = 16, B) Clinical Frailty Scale (CFS), n = 16 and C) gait speed (GS), n = 16. Pre-frailty thresholds indicated by the vertical broken lines; 1-2 on the FP, or 4-6 on the CFS, or a GS between ≥ 1.0 to < 1.5 meters per second (m/sec). The order of participants is consistent between each figure, in order to identify individual frailty scores across tools, as well as the change between time points. The arrow head indicates the direction and magnitude of change (right = negative or more frail; left = positive or less frail) from pre to post-intervention. Pre-intervention, nine, three, and thirteen participants were classified as pre-frail according to the FP, CFS, and GS, respectively. Pre-intervention, one participant was considered pre-frail according to all three tools, while seven participants were classified as pre-frail according to two tools. ■ = exercise (EX), ● = control (CON) group

Frailty: There was a time main effect for the FP (F (1,14) = 8.5, p ≤ 0.01, partial η2 = 0.4) and CFS (F (1,14) = 4.8, p ≤ 0.05, partial η2 = 0.3) but no group main effect (F (1, 14) = 1.423, p = 0.25, partial η2 = 0.09). Following the 12-week intervention, five and six participants of the EX were less frail according to the FP and CFS, respectively. Gait speed was faster for all EX participants. The CON became more frail according to the FP (n=1), CFS (n=2), and GS (n=8), respectively (Figure 2).

**Figure 3:**
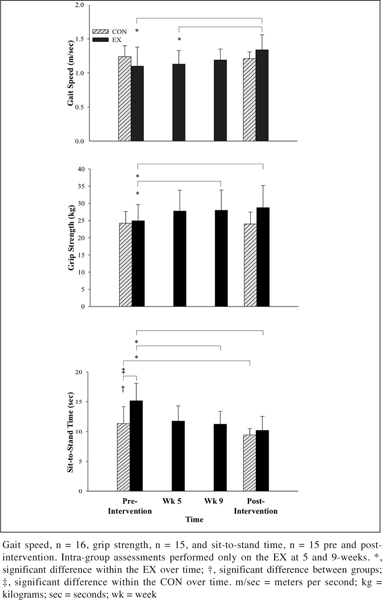
Control (CON) and Exercise (EX) group results for functional task measures

Functional Task Performance: EX demonstrated improvements in post-intervention GS (F (1, 7) = 15.2, p ≤ 0.01, partial η2 = 0.7) and grip strength (F (1, 6) = 17.3, p ≤ 0.01, partial η2 = 0.7; Figure 3). STS time (F (1, 13) = 4.8, p ≤ 0.05, partial η2 = 0.3) in the CON was faster pre-intervention (F (1, 13) = 6.6, p = 0.02, partial η2 = 0.3) but no significant difference existed post-intervention (F (1, 13) = 0.7, p = 0.4, partial η2 = 0.0) as a result of a significant improvement in the EX (5.0 sec, F (1, 6) = 18.2, p ≤ 0.01, partial η2 = 0.8).

**Figure 4:**
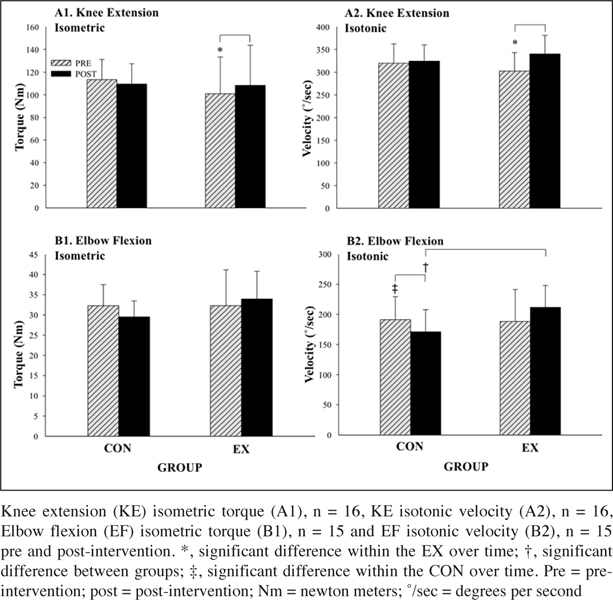
Control (CON) and Exercise (EX) group results for strength measures

Muscle Strength Performance: KE isometric torque (F (1, 7) = 5.9, p ≤ 0.05, partial η2 = 0.5) and isotonic velocity (F (1, 7) = 17.5, p = ≤ 0.01, partial η2 = 0.7) improved in the EX post- intervention (Figure 4). There was a significant interaction for EF isotonic velocity (F (1, 13) = 14.8, p ≤ 0.01, partial η2 = 0.5) as the CON was slower post-intervention (-20.2 °/sec, F (1, 7) = 21.7, p ≤ 0.01, partial η2 = 0.8).

## Discussion

Findings suggest that this intervention strategy appears to not only be feasible and safe for pre-frail females, but it can also improve frailty status, functional task performance, and strength measures. Conversely, CON participants became more frail over the same time period, showed little change in GS, grip and KE strength, and suffered a significant decline in EF isotonic strength.

This is the first study to include high-intensity, free-weight, functional resistance training in pre-frail older females, assessed via non-modified frailty tools pre and post-intervention. The lack of adverse events suggest that this style of training is safe when supervised and programmed correctly. The high adherence rates and lack of dropout may indicate a high degree of enjoyment. Previous interventions in pre-frail older adults have generally utilized high-repetition, low-intensity, singlejoint resistance training exercises, which, despite a lack of evidence, may be attributed to the commonly held belief that the alternative is unsafe (20).

Although frailty status was inconsistent between tools, we believe that by utilizing a combination of tools, the sample is reflective of the pre-frail/vulnerable population under investigation. For example, at baseline, our participant with the fastest gait speed was also considered pre-frail according to the FP. Frailty is a dynamic syndrome, as indicated by the different levels of frailty described across most assessment tools (25, 26), and there is growing support for disassociating frail from pre-frail (1, 5). Sex-differences also further complicate frailty (9,10). Previous research have reported a wide-range of values for the percentage (21-48%) of participants that have demonstrated an improvement in frailty status. Regardless of the tool, our percentage (> 63%) of participants that reversed frailty exceeds all other previous interventions that have included a pre-frail population, and measured frailty status pre and post-exercise intervention (15, 16, 17, 18, 19).

All measures of functional task performance consistently improved for the EX. Kwon and colleges (17) assessed both GS and grip strength, but only reported improvements in the latter (2.3 kg) for their intervention group, which was less than the 3.9 kg change observed in our EX. Improvements in functional task performance complement the observed changes in the Biodex measures.

The EX demonstrated significant improvement in KE isometric and isotonic strength post-intervention. Additionally, our EX maintained EF isotonic strength while the CON experienced a significant decline. Both Chan et al., (16) and Ng et al., (19) reported improvements in KE isometric strength in both their exercise and non-exercise groups postintervention, and therefore, exercise may not be responsible for improvement. Our study is the first to identify strength values for isotonic KE, as well as isometric and isotonic EF in prefrail older females, as part of an exercise intervention that used standard frailty assessment tools as both an inclusion criteria and outcome measure.

Synergistically (24), our study findings suggest that this intense resistance training program is not only safe for pre-frail females, but necessary to make enhanced improvements in frailty status, functional task performance, and muscle strength. However, this study is not without limitation. The sample size was relatively small but strength measures were appropriately powered to observe statistically meaningful changes. Moreover, the small group size likely positively influenced participants' adherence to the program (88.3%). Further work is needed to assess the factors that contribute to adherence in this population. Given the limited number of study personnel, the lead student investigator conducted the exercise training and was not blinded to group assignment but pre-intervention scores were unavailable at intra and post-intervention testing, and file names were coded during analysis. Despite the limitations, we believe the findings support undertaking a large randomized controlled trial.

## Conclusion

MCE inclusive of high-intensity, free-weight, functional resistance training is feasible and safe in pre-frail older females. When compared to previous studies that have measured prefrailty status as both an inclusion criteria and outcome measure, it appears to lead to greater improvements in frailty status, functional task performance, and muscle strength. A larger randomized controlled trial is required to confirm our findings.

## Funding

Partial funding for this study through the Canadian Institutes for Health Research (CIHR) Grant # 385692. The sponsors had no role in the design and conduct of the study; in the collection, analysis, and interpretation of data; in the preparation of the manuscript; or in the review or approval of the manuscript.

## Ethics approval and consent

All participants read and signed a letter of informed consent. Ethical approval was granted by the institutional Research Ethics Board (H16-00712).

## Competing interests

None

## Trial Registration

This study was prospectively registered with ClincalTrials.gov (NCT02952443) on October 31, 2016.

## Availability of the data and materials

The original data and materials are available through the institutions open access graduate thesis repository https://open.library.ubc.ca/cIRcle/collections/ubctheses/24/items/1.0353165
